# Agent orange exposure and prostate cancer risk in the million veteran program

**DOI:** 10.2340/1651-226X.2024.25053

**Published:** 2024-05-23

**Authors:** Meghana S. Pagadala, Asona J. Lui, Allison Y. Zhong, Julie A. Lynch, Roshan Karunamuni, Kyung Min Lee, Anna Plym, Brent S. Rose, Hannah K. Carter, Adam S. Kibel, Scott L. DuVall, J. Michael Gaziano, Matthew S. Panizzon, Richard L. Hauger, Tyler M. Seibert

**Affiliations:** a Research Service, VA San Diego Healthcare System, San Diego, CA, USA; b Medical Scientist Training Program, University of California San Diego, La Jolla, CA, USA; c Biomedical Science Program, University of California San Diego, La Jolla, CA, USA; d Department of Radiation Medicine and Applied Sciences, University of California San Diego, La Jolla, CA, USA; e VA Informatics and Computing Infrastructure (VINCI), VA Salt Lake City Health Care System, Salt Lake City, UT, USA; f Department of Internal Medicine, Division of Epidemiology, University of Utah School of Medicine, Salt Lake City, UT, USA; g Division of Urology, Brigham and Women’s Hospital, Boston, MA, USA; h Epidemiology, TH Chan School of Public Health, Harvard University, Boston, MA, USA; i Department of Medical Epidemiology and Biostatistics, Karolinska Institutet, Stockholm, Sweden; j Department of Medicine, University of California San Diego, La Jolla, CA, USA; k Massachusetts Veterans Epidemiology Research and Information Center (MAVERIC), VA Boston Healthcare System, Boston, MA, USA; l Department of Medicine, Brigham and Women’s Hospital, Boston, MA, USA; m Center for Behavioral Genetics of Aging, University of California San Diego, La Jolla, CA, USA; n Center of Excellence for Stress and Mental Health (CESAMH), VA San Diego Healthcare System, San Diego, CA, USA; o Department of Radiology, University of California San Diego, La Jolla, CA, USA; p Department of Bioengineering, University of California San Diego, La Jolla, CA, USA

**Keywords:** Agent Orange, prostate cancer, MVP, race/ethnicity, health disparities

## Abstract

**Background:**

The US government considers veterans to have been exposed to Agent Orange if they served in Vietnam while the carcinogen was in use, and these veterans are often deemed at high risk of prostate cancer (PCa). Here, we assess whether presumed Agent Orange exposure is independently associated with increased risk of any metastatic or fatal PCa in a diverse Veteran cohort still alive in the modern era (at least 2011), when accounting for race/ethnicity, family history, and genetic risk.

**Patients and Methods:**

Participants in the Million Veteran Program (MVP; enrollment began in 2011) who were on active duty during the Vietnam War era (August 1964-April 1975) were included (*n* = 301,470). Agent Orange exposure was determined using the US government definition. Genetic risk was assessed via a validated polygenic hazard score. Associations with age at diagnosis of any PCa, metastatic PCa, and death from PCa were assessed via Cox proportional hazards models.

**Results and Interpretation:**

On univariable analysis, exposure to Agent Orange was not associated with increased PCa (hazard ratio [HR]: 1.02, 95% confidence interval [CI]: 1.00–1.04, *p* = 0.06), metastatic PCa (HR: 0.98, 95% CI: 0.91–1.05, *p* = 0.55), or fatal PCa (HR: 0.94, 95% CI: 0.79–1.09, *p* = 0.41). When accounting for race/ethnicity and family history, Agent Orange exposure was independently associated with slightly increased risk of PCa (HR: 1.06, 95% CI: 1.04–1.09, <10^-6^) but not with metastatic PCa (HR: 1.07, 95% CI: 0.98–1.15, *p* = 0.10) or PCa death (HR: 1.02, 95% CI: 0.83–1.23, *p* = 0.09). Similar results were found when accounting for genetic risk. Agent Orange exposure history may not improve modern PCa risk stratification.

## Introduction

Agent Orange, a mixture of herbicides 2,4-dichlorophenoxyacetic acid (2,4-D) and 2,4,5-Trichlorophenoxyacetic acid (2,4,5-T), kerosene, and diesel fuel, was used in the Vietnam War to clear dense vegetation and destroy food crops. A potential association between 2,4-D and 2,4,5-T exposure [1–3] increased the risk of non-Hodgkin lymphoma, soft-tissue sarcoma, and bladder and lung cancers and has been debated since the 1980s [4], though no adequate epidemiological evidence has supported that conclusion [5]. Agent Orange and early formulations of 2,4-D and 2,4,5-T, were contaminated with a dioxin compound known as 2,3,7,8-tetrachlorobenzo-p-dioxin (TCDD), which has been classified as a carcinogen since the 1990s. The Agent Orange Act of 1991 defines exposure to include all veterans who served anywhere in Vietnam between January 9, 1962 to May 7,1975; this Federal definition is used to guide current preventive healthcare policies in this population [6–8]. In the 2000s, a potential association was acknowledged between Agent Orange exposure and genitourinary cancers [9]. However, evidence linking Agent Orange exposure to increased PCa risk or associated mortality among Vietnam War Veterans has been limited to small case series [10–16]. These small studies have found Agent Orange to be associated with slightly lower age at PCa diagnosis, higher incidence of Stage IV disease, and lower rates of biochemical control [8, 16].

We investigated the association between Agent Orange exposure and PCa risk in the VA Million Veteran Program (MVP), a population-based cohort that started enrollment in 2011 with genotyping, long-term follow-up, and linked clinical records for over 870,000 participating US veterans. The MVP is one of the largest and most diverse electronic health record-linked biobanks in the world, with a unique structure that allows for detailed investigation into the interactions between inherited risk and Agent Orange exposure in US veterans [17]. We tested the hypothesis that Agent Orange exposure, using the practical government definition, is associated with PCa outcomes and thus might improve modern PCa risk stratification for early detection strategies. Moreover, as MVP data have the potential to inform future clinical care and clinical trials (e.g. NCT05129605), it is important to understand how Agent Orange exposure might influence results in this population.

## Methods

### Participants

We obtained data from MVP for individuals recruited from 63 VA Medical Centers across the United States (US) beginning in 2011. All veterans were eligible for participation in MVP. Study participation included consenting to access the participant’s electronic health records for research purposes. The MVP received ethical and study protocol approval from the VA Central Institutional Review Board in accordance with the principles outlined in the Declaration of Helsinki. We limited the present study to males on active duty during the Vietnam War era (August 1964–April 1975) (Table 1). We included PCa diagnoses at any point after Vietnam War service, regardless of when the participant enrolled in MVP. At the time of MVP enrollment, 265,146 participants had no known PCa, 22,609 had a non-metastatic PCa diagnosis, and 1,218 had been diagnosed with metastatic PCa.

**Table 1:** Participant characteristics for self-reported race/ethnicity groups among MVP participants who served on active duty during the Vietnam War era (August 1964–April 1975).

	All	Self-reported Race/Ethnicity							
		Non-Hispanic White	Black or African American	Hispanic White	Asian	Native American	Pacific Islander	Other	Unknown
Active duty during Vietnam War	301,470 (84,326)	230,506 (68,171)	45,257 (9,216)	11,009 (3,176)	1,915 (447)	3,082 (860)	1,292 (339)	4,155 (1,096)	4,254 (1,021)
Fatal prostate cancer	795 (221)	525 (155)	200 (50)	27 (6)	<10 (1)	<10 (1)	<10 (1)	13 (2)	23 (5)
Metastatic prostate cancer	3,828 (1,113)	2,495 (818)	1,033 (219)	125 (35)	23 (4)	29 (8)	12 (4)	47 (13)	64 (12)
Any prostate cancer	42,569 (12,822)	29,482 (9,555)	10,084 (2,385)	1,278 (403)	224 (45)	366 (130)	152 (46)	481 (135)	501 (123)

Numbers indicate participants available for analysis. Numbers in parentheses indicate participants with Agent Orange exposure.

### Potential Agent Orange exposure

Potential exposure to Agent Orange was determined by the VA Compensation & Pension Committee, as recorded in the MVP data core. As per the legal US government definition, veterans who served physically (on land or inland waterways) in Vietnam during periods of Agent Orange use by the US military were considered exposed to Agent Orange (January 9, 1962–May 7, 1975). Information about the intensity (amount and duration) of Agent Orange exposure for each individual is not known, consistent with routine clinical reality.

### Clinical data extraction

PCa diagnosis, age at diagnosis, prostate-specific antigen (PSA) tests, and date of last follow-up were retrieved from the VA Corporate Data Warehouse based on ICD codes and VA Central Cancer Registry data. Age at diagnosis of metastatic PCa indicated the age of the participant when diagnosed with either nodal or distant metastases as determined through a validated natural language processing tool [18]. Fatal PCa information was determined from National Death Index. Participants with ICD10 code ‘C61’ as underlying cause of death were considered to have died from PCa. Family history was recorded as either the presence or absence of one or more first-degree relatives with PCa. Among the participants eligible for analysis, over 99% had received at least one PSA test in the VA system, though the age at testing and frequency of testing were variable, and clinical indications (screening vs. diagnostic workup) are not known.

### Genetic risk: Polygenic Hazard Score (PHS290)

Blood sampling, DNA extraction, quality controls, and imputation were conducted by MVP as described previously [9, 14]. The MVP 1.0 genotyping array contains a total of 723,305 variants, enriched for low-frequency variants in African and Hispanic populations and variants associated with diseases common to the VA population [16].

To assess genetic risk, we calculated a previously developed and validated polygenic hazard score using 290 common genetic variants (PHS290) that reliably stratifies men for age-dependent genetic risk of PCa and is associated with PCa, metastatic PCa, and PCa death [18–20]. Details of PHS290 calculation in MVP are described elsewhere [18, 19]. PHS290 performs well in diverse datasets and is independently associated with PCa risk [18, 19].

### Cox proportional hazards analysis

We used Cox proportional hazards models to evaluate the association of Agent Orange exposure with three clinical endpoints: age at diagnosis of PCa, age at diagnosis of metastatic PCa, and age at death from PCa. We also analyzed self-reported racial/ethnic subgroups. Participants with both Black race and Hispanic ethnicity were included in a single category for Black or African American race. Where individuals did not meet the endpoint of interest, we censored at age at last follow-up.

To assess for independent association of Agent Orange exposure with PCa endpoints, we used multivariable Cox proportional hazards models with race/ethnicity, family history, and PHS290. For race/ethnicity hazard ratios (HRs), we used Non-Hispanic White as reference. For PHS290, we illustrated the effect size via the HR for the highest 20% versus lowest 20% of genetic risk (HR80/20) and between other strata of PHS290. These percentiles refer to previously defined absolute thresholds of PHS290 [18, 19]. We assessed statistical significance with two-tailed alpha at 0.01.

### PSA testing

Screening has been shown in a large, randomized trial to increase PCa incidence and reduce cause-specific mortality [22], raising the possibility that PSA testing may confound any impact of Agent Orange exposure. We ascertained the number of PSA tests each participant underwent and evaluated associations between Agent Orange exposure and number of pre-diagnostic PSA tests (≥2 years prior to PCa diagnosis) via linear regression. Multivariable linear regressions used race/ethnicity, family history, and PHS290 as predictive variables in addition to Agent Orange exposure.

## RESULTS

We found 301,470 veterans eligible for this analysis. Median age at PCa diagnosis was 65.3 years [interquartile range (IQR): 61–69]. Median age at last follow-up was 71.3 [68–74].

**Figure 1:**
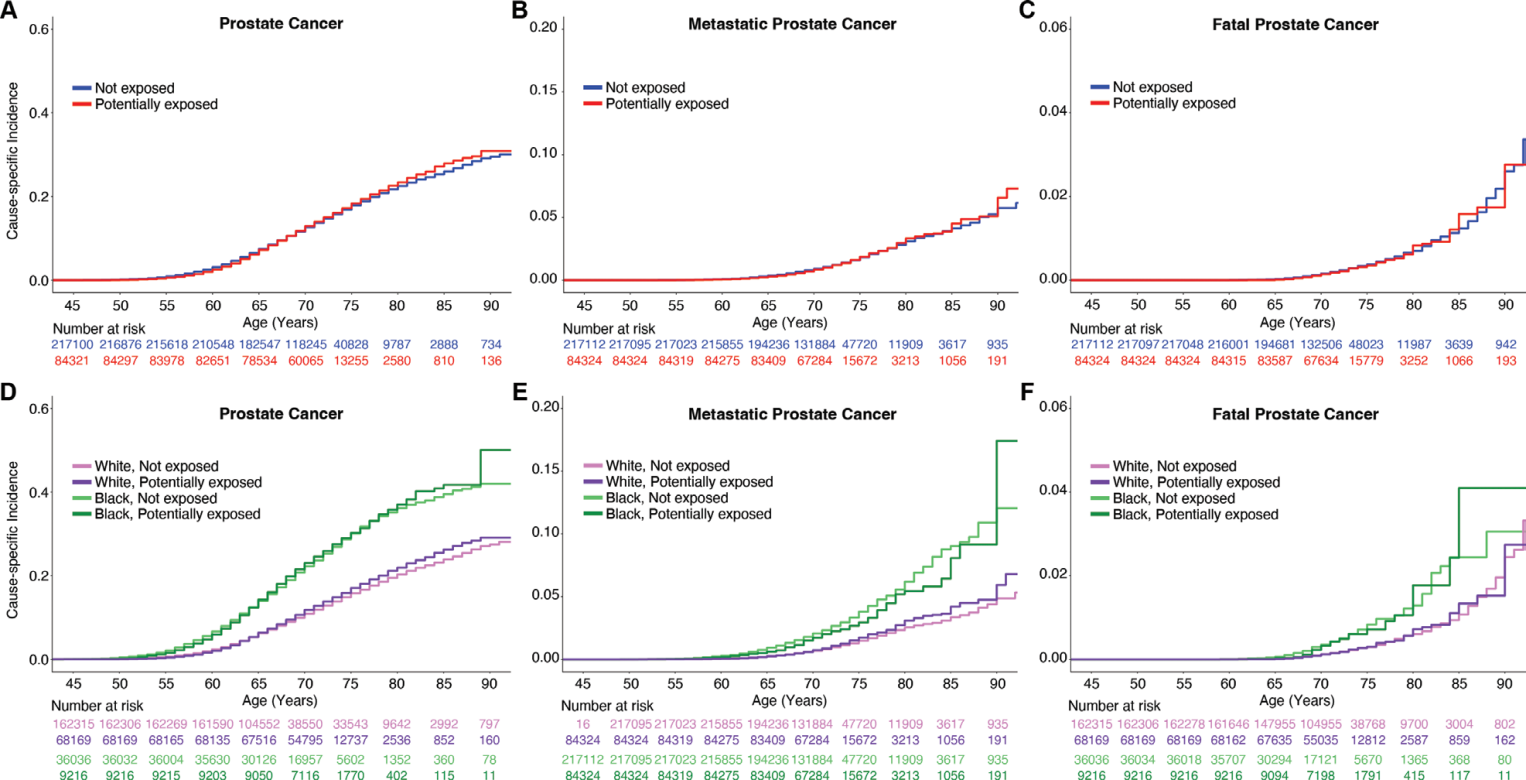
Million Veteran Program (MVP) cause-specific cumulative incidence based on Agent Orange exposure. Cause-specific cumulative incidence among MVP participants on active duty during the Vietnam War, stratified by Agent Orange exposure status (top row) and stratified by self-reported race (bottom row) for (A, D) all prostate cancer, (B, E) metastatic prostate cancer, and (C, F) fatal prostate cancer. ‘White’ indicates Non-Hispanic White participants, and ‘Black’ indicates Black and Hispanic Black participants.

On univariable analysis, Agent Orange exposure was not associated with increased PCa diagnosis (HR: 1.02, 95% confidence interval [CI]: 1.00–1.04, *p* = 0.06) (Figure 1; Supplemental Table 1). Some statistically significant associations were found in subgroups based on race and ethnicity (Supplemental Table 1). In the Non-Hispanic White group, Agent Orange exposure was associated with increased PCa (HR: 1.08, 95% CI: 1.05–1.10, *p* < 10^-8^) and metastatic PCa diagnosis (HR: 1.13, 95% CI: 1.03–1.22, *p* < 10^-2^). A statistically significant association in the opposite direction was observed in the Black or African American group: those with Agent Orange exposure were somewhat less likely to develop PCa (HR: 0.82, 95% CI: 0.71–0.95, *p* < 10^-2^). No evidence of association with fatal PCa was seen in MVP participants. Cause-specific cumulative incidence curves for PCa were qualitatively similar regardless of Agent Orange exposure status (Figure 1).

When accounting for race/ethnicity and family history, Agent Orange exposure was an independent risk factor for PCa diagnosis (HR: 1.06, 95% CI: 1.04–1.09, *p* < 0.05) but not for metastatic PCa or PCa death. (Table 2). Genetic risk (PHS290) was strongly associated with all PCa endpoints, but accounting for this genetic effect had no impact on the association between Agent Orange exposure and PCa diagnosis (Supplemental Table 2). Agent Orange exposure did not differentially modulate PCa risk among men with high genetic risk (PHS290 >80^th^ percentile, as defined previously [21]) or across any PHS290 values (Supplemental Table 3, Supplemental Figure 1).

**Table 2:** Multivariable models combining self-reported race/ethnicity, family history, and Agent Orange exposure for three PCa clinical endpoints.

Clinical Endpoint	Self-Reported Race/Ethnicity							Family History	Agent Orange Exposure
	Black or African American	Hispanic White	Asian	Native American	Pacific Islander	Unknown	Other		
Fatal Prostate Cancer	2.34 [1.87–2.88]**	1.06 [0.53–1.6]	0.26 [0.0–0.8]	0.77 [0.0–1.86]	NA	NA	2.02 [1.04–3.17]	1.89 [1.45–2.34]*	1.02 [0.83–1.23]
Metastatic Prostate Cancer	2.49 [2.26–2.72]***	1.24 [0.97–1.52]	1.03 [0.55–1.53]	0.98 [0.52–1.49]	0.56 [0.0–1.42]	1.78 [0.77–3.13]	1.44 [1.06–1.86]	1.51 [1.34–1.7]**	1.07 [0.98–1.17]
Prostate Cancer	2.2 [2.13–2.26]***	1.02 [0.95–1.1]	0.87 [0.75–1.0]	1.02 [0.9–1.14]	0.89 [0.62–1.18]	0.83 [0.62–1.05]	1.05 [0.95–1.15]	1.85 [1.79–1.92]***	1.06 [1.04–1.09]*

Cox proportional hazards results for association with age at death from PCa, age at diagnosis of metastatic PCa, and age at diagnosis with PCa. *P*-values reported are from multivariable models using self-reported race/ethnicity, family history, and Agent Orange exposure (yes or no). Hazard ratios for race/ethnicity were estimated using Non-Hispanic White as the reference. Hazard ratios for family history were for one or more first-degree relatives diagnosed with prostate cancer. This multivariable analysis was limited to the 213,856 participants who were on active duty during the Vietnam War and for whom family history information was available. Numbers in brackets are 95% confidence intervals. Significant predictors in the multivariable model are indicated by

* (*p* < 0.01),

** (*p* < 10^-10^), and

*** (*p* < 10^-16^).

On univariable and multivariable linear regression analyses in this population, there was no evidence of association between Agent Orange exposure and increased screening. Agent Orange exposure was associated with a statistically significant but small reduction in screening intensity on univariable analysis – 8.3 PSA tests compared to 9 PSA tests for those not exposed. On the other hand, self-reported Black race was associated with increased PSA testing, concordant with guidelines that support stronger consideration of screening for men at higher risk [20] (Supplemental Table 4).

## DISCUSSION

In a large, diverse, population-based cohort of US Veterans who served during the Vietnam War and were still alive to enroll in MVP in 2011, Agent Orange exposure was weakly associated with overall PCa, but not metastatic or fatal PCa. Importantly, we present the first multivariable analysis in a population-based cohort to assess whether Agent Orange exposure was an *independent* risk factor for PCa outcomes when accounting for family history, ancestry, and/or genetic risk. Our findings may have pragmatic implications for early detection strategies and suggest the US definition of Agent Orange exposure does not substantially increase risk of morbidity or mortality from PCa, at least for individuals alive today. Also, this study helps inform inclusion criteria for clinical trial enrollment in the VA and sets the foundation to better understand veteran exposures such as burn pits that need to be monitored

Details confirming actual Agent Orange exposure including duration or intensity are not available in MVP or routine clinical practice. Some veterans who physically served in Vietnam while Agent Orange was in use may have had heavy and/or frequent exposure, whereas others may have escaped with little to no exposure. It is possible that intense Agent Orange exposure is associated with aggressive PCa, though adequate data will likely never be available to answer this question. The definition of Agent Orange exposure used in this study is also used by the VA Compensation & Pension Committee to address the needs of potentially exposed individuals. Use of this definition estimates associations of the *average* exposure by those veterans serving in Vietnam during use of Agent Orange. Among Veterans surviving to 2011 or later, we can conclude that average Agent Orange exposure among US veterans serving during Vietnam War era has a much smaller effect size than do family history, Black race, or high polygenic risk. On multivariable analysis, potential Agent Orange exposure yielded HRs < 1.10 for all PCa endpoints underscoring the fact that these statistical associations are not likely clinically meaningful, whereas HRs for metastatic PCa were 1.37 for family history, 1.97 for Black race, and 4.42 for individuals with high versus low polygenic risk (PHS290). Notably, effects may be underestimated as our study focused on veterans who were alive for MVP enrollment in 2011 and did not include veterans who may have died prior to 2011 from Agent Orange exposure effects.

Statistically significant associations in subgroup analyses of self-reported race/ethnicity were small and in opposite directions (increased risk after Agent Orange exposure for Non-Hispanic White participants and decreased risk for Black or African American participants). We interpret these subgroup findings cautiously. On the whole, there is not a clear and strong association of Agent Orange exposure and poor PCa outcomes in MVP.

This study was conducted using data from MVP, so the results may not be generalizable beyond the VA population. Potential differences in PCa screening intensity between exposure groups were not completely accounted for, though there was no evidence of increased PSA testing among those exposed to Agent Orange in this study. As sequencing for rare pathogenic mutations was not performed, it was also not possible to assess the impact of Agent Orange exposure on risk arising from, for example, germline *BRCA2* mutations, considering Agent Orange mutates genes and induces chromosomal aberrations.

## Author contributions

A.J.L., M.S.P., T.M.S. conceived and designed the analysis; A.J.L., M.S.P. and R.K. performed the analysis; J.A.L., K.M.L., S.L.D., J.M.G. contributed data and analysis tools; A.J.L., M.S.P., and T.M.S. wrote the paper with assistance from A.Y.Z., A.P., B.S.R., H.K.C., A.S.K., M.S.P., R.L.H.

## Supplementary Material





## Data Availability

It is not possible for the authors to directly share the individual-level data that were obtained from the MVP due to constraints stipulated in the informed consent. Anyone wishing to gain access to this data should inquire directly to MVP at MVPLOI@va.gov. The data generated from our analyses are included in the manuscript main text, tables, and figures.
