# Supplementary material for: Agent orange exposure and prostate cancer risk in the million veteran program
Source: Acta Oncol. 2024 May 23;63:25053. doi: 10.2340/1651-226X.2024.25053 (PMC11332552; doi:10.2340/1651-226X.2024.25053)
Supplement: Supplementary file 1 [file AO-63-25053-s1.pdf]

Supplementary material has been published as submitted. It has not been copyedited or typeset by Acta Oncologica.

Supplemental Table 1: Univariable association of Agent Orange exposure with any, metastatic, and fatal PCa. Hazard ratios (HR) from Cox Proportional Hazards model of Agent Orange exposure with age at prostate cancer, metastatic prostate cancer (nodal or distant), and death from prostate cancer. *P*-values reported are from univariable models using Agent Orange exposure (yes or no) as the sole predictor variable. Significant associations are indicated by *. NA: indicates subgroups for which there were insufficient participants to estimate results. n: indicates number at risk.

Race/Ethnicity	Any Prostate Cancer		Metastatic Prostate Cancer		Fatal Prostate Cancer	
	HR	P	HR	P	HR	P
**All** (n=301,470)	1.02 [1.00-1.04]	0.06	0.98 [0.91-1.05]	0.55	0.94 [0.79-1.09]	0.41
**Non-Hispanic White** (n=230,506)	**1.08 [1.05-1.10]**	**<10^{-8}***	**1.13 [1.03-1.22]**	**<10^{-2}***	1.01 [0.84-1.21]	0.88
**Black or African American** (n=45,257)	1.03 [0.98-1.08]	0.26	**0.82 [0.71-0.95]**	**<10^{-2}***	0.94 [0.68-1.27]	0.69
**Hispanic White** (n=11,009)	1.03 [0.91-1.15]	0.66	0.86 [0.58-1.24]	0.46	0.63 [0.17-1.46]	0.32
**Asian** (n=1,915)	0.74 [0.52-0.98]	0.07	0.65 [0.14-1.66]	0.42	NA	NA
**Native American** (n=3,082)	**1.26 [1.01-1.56]**	**0.04***	0.82 [0.32-1.76]	0.63	NA	NA
**Pacific Islander** (n=1,292)	1.05 [0.74-1.46]	0.80	1.26 [0.23-3.91]	0.71	NA	NA

**Other**<br>(n=4,155)	0.92 [0.75-1.12]	0.41	0.89 [0.41-1.65]	0.71	0.41 [0.0-1.28]	0.24
**Unknown**<br>(n=4,254)	0.96 [0.79-1.18]	0.70	0.66 [0.3-1.11]	0.19	0.85 [0.17-2.04]	0.76

Supplemental Table 2: Multivariable models combining self-reported race/ethnicity, family history, genetic risk, and Agent Orange exposure for three prostate cancer (PCa) clinical endpoints. Cox proportional hazards results for association with age at death from PCa, age at diagnosis of metastatic PCa, and age at diagnosis with PCa. *P*-values reported are from multivariable models using self-reported race/ethnicity, family history, genetic risk (PHS290), and Agent Orange exposure (yes or no). For PHS290, effect size was illustrated via the hazard ratio ($HR_{80/20}$) for the highest 20% vs. lowest 20% of genetic risk. Hazard ratios for race/ethnicity were estimated using Non-Hispanic White as the reference. Hazard ratios for family history were for one or more first-degree relatives diagnosed with prostate cancer. This multivariable analysis was limited to the 213,856 participants who were on active duty during the Vietnam War and for whom family history information was available. Numbers in brackets are 95% confidence intervals. Significant predictors in the multivariable model are indicated by *($p<0.01$), **($p<10^{-10}$) and ***($p<10^{-16}$).

Clinical Endpoint	Self-Reported Race/Ethnicity							Family History	PHS290	Agent Orange Exposure
	Black or African American	Hispanic	Asian	Native American	Pacific Islander	Unknown	Other		$HR_{80/20}$	
**Fatal PCa**	**1.83 [1.45-2.28]***	1.09 [0.54-1.65]	0.36 [0.0-1.16]	0.74 [0.0-1.8]	NA	NA	1.98 [1.03-3.14]	**1.7 [1.29-2.12]***	**4.88 [3.83-6.17]*****	1.01 [0.83-1.22]
**Metastatic PCa**	**1.97 [1.78-2.16]*****	1.27 [0.99-1.56]	1.4 [0.74-2.1]	0.94 [0.5-1.42]	0.54 [0.0-1.37]	1.87 [0.81-3.28]	1.42 [1.04-1.84]	**1.37 [1.21-1.54]***	**4.42 [3.97-4.94]*****	1.07 [0.98-1.17]
**PCa**	**1.72 [1.66-1.77]*****	1.05 [0.98-1.13]	1.2 [1.04-1.38]	0.97 [0.86-1.09]	0.86 [0.6-1.13]	0.86 [0.65-1.08]	1.04 [0.95-1.14]	**1.69 [1.63-1.75]*****	**4.91 [4.74-5.07]*****	**1.06 [1.04-1.09]***

Supplemental Table 3: Multivariable models combining self-reported race/ethnicity, family history, and Agent Orange exposure for three prostate cancer (PCa) clinical endpoints in participants with high genetic risk. Cox proportional hazards results for association with age at death from PCa, age at diagnosis of metastatic PCa, and age at diagnosis with PCa in men with PHS290 score > 80th percentile. *P*-values reported are from multivariable models using self-reported race/ethnicity, family history, and Agent Orange exposure (yes or no). Hazard ratios for race/ethnicity were estimated using Non-Hispanic White as the reference. Hazard ratios for family history were for one or more first-degree relatives diagnosed with prostate cancer. This multivariable analysis was limited to the 54,841 participants with PHS290 > 80th percentile (i.e., high genetic risk) who were on active duty during the Vietnam War and for whom family history information was available. Numbers in brackets are 95% confidence intervals. Significant predictors in the multivariable model are indicated by *(p<0.01), **($p<10^{-10}$) and ***($p<10^{-16}$).

Clinical Endpoint	Self-Reported Race/Ethnicity							Family History	Agent Orange Exposure
	Black or African American	Hispanic	Asian	Native American	Pacific Islander	Unknown	Other		
**Fatal PCa**	**1.54 [1.13-2.08]***	0.83 [0.18-1.68]	NA	1.0 [0.0-2.72]	NA	NA	1.82 [0.58-3.48]	1.4 [0.93-2.0]	0.99 [0.74-1.27]
**Metastatic PCa**	**1.77 [1.56-2.02]****	1.16 [0.75-1.62]	0.69 [0.0-1.86]	0.87 [0.32-1.58]	1.27 [0.0-3.27]	0.52 [0.0-1.85]	1.21 [0.69-1.8]	**1.41 [1.18-1.68]***	1.01 [0.89-1.15]
**PCa**	**1.55 [1.49-1.61]*****	0.98 [0.87-1.09]	1.29 [0.96-1.69]	0.96 [0.81-1.15]	0.69 [0.37-1.08]	1.02 [0.67-1.42]	1.05 [0.91-1.19]	**1.70 [1.63-1.79]*****	**1.07 [1.04-1.12]***

Supplemental Table 4: Univariable and multivariable linear regressions assessing association between Agent Orange exposure, self-reported race/ethnicity, genetic risk, and family history with screening intensity among Veterans who had served on active duty in the Vietnam War era. Screening intensity was estimated by the total number of pre-diagnostic PSA tests performed for a participant. 'Pre-diagnostic' refers to any test performed >2 years prior to diagnosis of prostate cancer in affected individuals or any PSA test performed in a participant never diagnosed with prostate cancer. Results are for association between each factor and the number of PSA tests recorded in the VA medical record for a given participant. Coefficients for race/ethnicity were estimated using Non-Hispanic White as reference. Family history was defined as one or more first-degree relatives diagnosed with prostate cancer. Numbers in brackets are 95% confidence intervals. Significant predictors in each model are indicated by *(p<0.01), **($p<10^{-10}$) and ***($p<10^{-16}$). On univariable analysis, Agent Orange exposure was associated with an average of 0.73 fewer PSA tests in this population than performed in men not exposed to Agent Orange.

Univariable		Multivariable										
Intercept	Agent Orange Exposure	Intercept	Self-Reported Race/Ethnicity							Family History	PHS290	Agent Orange Exposure
			Black	Hispanic White	Asian	Native American	Pacific Islander	Other	Unknown		score	
9.00 [8.99-9.01]***	-0.73 -[0.76-0.71]***	8.69 [8.67-8.70]***	+1.13 [1.08-1.18]***	+0.51 [0.42-0.68]*	-1.55 -[1.73-1.37]***	+1.04 [0.89-1.18]**	-0.28 [-0.64-0.07]	+0.61 [0.50-0.72]*	-2.04 -[2.34-1.74]**	-0.34 -[0.40-0.29]*	-0.42 -[0.41-0.44]***	-0.82 -[0.85-0.79]***
