# Supplementary material for: Agent orange exposure and prostate cancer risk in the million veteran program
Source: Acta Oncol. 2024 May 23;63:25053. doi: 10.2340/1651-226X.2024.25053 (PMC11332552; doi:10.2340/1651-226X.2024.25053)

Supplementary material has been published as submitted. It has not been copyedited or typeset by Acta Oncologica

Supplemental Figure 1. Million Veteran Program (MVP) cause-specific cumulative incidence based on Agent Orange exposure for participants with high (top 20%) genetic risk of prostate cancer. Cause-specific cumulative incidence among MVP participants on active duty during the Vietnam War, stratified by Agent Orange exposure and PHS290 for **(A)** all prostate cancer, **(B)** metastatic prostate cancer, and **(C)** fatal prostate cancer. 'High PHS290' indicates participants with PHS in the top 20% of genetic risk, as assessed by PHS290, a polygenic hazard score.

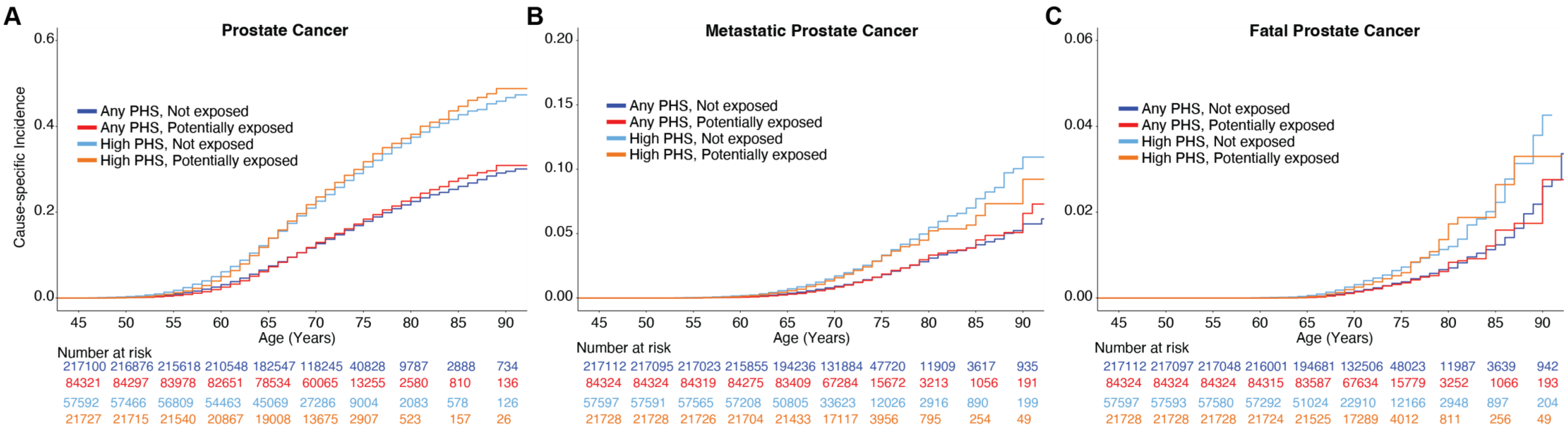

Supplement: Supplementary file 2 [file AO-63-25053-s2.pdf]
